# A Self-Healing and Shape Memory Polymer that Functions at Body Temperature

**DOI:** 10.3390/molecules24183224

**Published:** 2019-09-04

**Authors:** Hui-Ying Lai, Hong-Qin Wang, Jian-Cheng Lai, Cheng-Hui Li

**Affiliations:** State Key Laboratory of Coordination Chemistry, School of Chemistry and Chemical Engineering, Nanjing National Laboratory of Microstructures, Collaborative Innovation Center of Advanced Microstructures, Nanjing University, Nanjing 210023, China

**Keywords:** self-healing, PDMS, hydrogen bonding, shape memory

## Abstract

Dual-functional polymeric system combining shape memory with self-healing properties has attracted increasingly interests of researchers, as both of these properties are intelligent and promising characteristics. Moreover, shape memory polymer that functions at human body temperature (37 °C) are desirable because of their potential applications in biomedical field. Herein, we designed a polymer network with a permanent covalent crosslinking and abundant weak hydrogen bonds. The former introduces elasticity responsible and maintain the permanent shape, and the latter contributes to the temporary shape via network rearrangement. The obtained PDMS-COO-E polymer films exhibit excellent mechanical properties and the capability to efficiently self-heal for 6 h at room temperature. Furthermore, the samples turn from a viscous state into an elastic state at 37 °C. Therefore, this polymer has shape memory effects triggered by body temperature. This unique material will have a wide range of applications in many fields, containing wearable electronics, biomedical devices, and 4D printing.

## 1. Introduction

Most natural biomaterials have an intrinsic ability to self-heal upon encountering damages. To mimic the self-healing properties of natural biomaterials and prolong the lifetime of the material in various applications, many synthetic self-healing polymers which can repair the internal or external damages have been developed [1,2,3,4]. The initial polymeric self-healing materials relied on micro-encapsulated healing agents within the bulk polymer [5,6,7,8,9]. Due to the consumption of the encapsulated agents, the repair in such system is not repeatable. To solve this problem, reversible covalent bonds (such as Diels-Alder (DA) reactions [10,11], [2 + 2] cycloaddition [12], acylhydrazone bonds [13], trithiocarbonate units [14], disulfide moieties [15], and diarylbibenzofuranone [16]) or non-covalent interactions (such as hydrogen bonding [17,18], hydrophobic associations [19], π–π stacking [20], ionic interactions [21], and metal-ligand interactions [22,23]) are incorporated into polymers as cross-linkages so that they will break and reform reversibly, leading to repeatable self-healing.

On the other hand, shape-memory polymers (SMPs), which are a group of polymers that have the capability of recovering their permanent shape upon the application of an external stimulus, are also the main concern of materials scientists, as such polymers possess a broad range of applications that changes from deployable structures and actuators to biomedical devices as well as sensors. So far, SMPs that are triggered by various external stimulus, such as heat [24,25,26], ultraviolet irradiation [27], electricity [28,29,30], water or moisture [31], and magnetic field [32], have been reported.

Recently, dual-functional polymeric system combining shape memory with self-healing properties has attracted increasingly interests of researchers [33,34,35,36,37,38,39,40]. Sodano presented polyurethanes based on Diels-Alder (DA) reaction exhibiting shape memory behavior and self-healing capabilities [33]. Wang also constructed a polymer network possessing both shape recovery and self-healing properties upon utilizing the cross-linking reaction between poly(vinyl butyral) (PVB) and hexamethylene diisocyanate (HDI) [34]. Both of them demonstrated that traditional materials can exhibit SME as well as self-healing properties after chemical modifications. Such dual-functional polymeric systems present potential to broaden and enrich the developments of smart materials and structures [36]. Moreover, shape memory effect has been shown to improve the self-healing property by closing mechanical damage or crack. For example, Mather and coworkers reported a new strategy of healing mechanism based on shape memory assisted self-healing polymers (SMASH), which are contributed to diffusion and re-bonding of polymer chain at damage areas above *T_g_* [37].

For applications in the biomedical field, such as clot removal devices [41], surgical sutures [42], dialysis needles [43], orthopedic suture anchors [44,45], and vascular stents [46], the trigger temperature of SMP’s should be near the human body temperature (37 °C). However, the precise tuning of the thermal transition temperature of SMP’s is highly challenging. So far, only a few SMP’s that function at body temperature have been reported. Anthamatten et al. developed a body temperature triggered poly(caprolactone) SMP networks by inclusion of non-crystallizable molecular linkers and by variation of prepolymer molecular weight [47]. Jiang et al. reported biodegradable body temperature-responsive shape memory polyurethanes, with self-healing function, which were synthesized from polycarbonate diol (PCDL), Methylene diphenyl diisocyanate (MDI) and different chain extenders [48]. Acar et al. synthesized polysulfone-based SMPs with tunable transition temperature via atom transfer radical polymerization. By altering the topology and the ratio of soft/hard segments, the triggering temperature can be tuned to 32.7–39.1 °C [49].

In this work, we designed and synthesized a self-healing and shape memory polymer that functions at body temperature. This polymer (denoted as PDMS-COO-E) was obtained by crosslinking a PDMS polymer containing dense carboxylate groups (100% mol) (PDMS-COOH) with small amount of poly(ethylene glycol) diglycidyl ether (PEGDGE). The PDMS-COO-E polymer films are highly stretchable (strain >500%), can autonomously self-heal at room temperature, and have shape memory effects triggered by body temperature. This unique material will have a wide range of applications in many fields, containing wearable electronics, biomedical devices and 4D printing.

## 2. Results and Discussion

### 2.1. Synthesis and General Characterization

**Figure 1:**
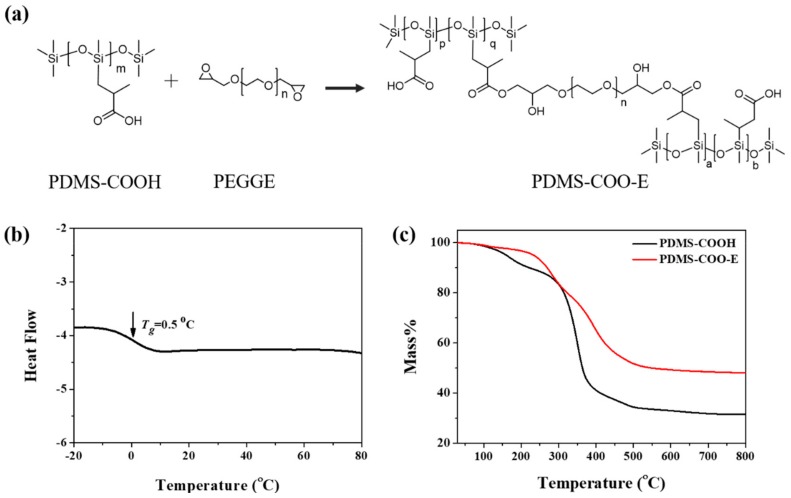
(**a**) Synthesis route of PDMS-COO-E polymer. (**b**) The DSC curve of PDMS-COO-E polymer. (**c**) The TGA curves of PDMS-COOH and PDMS-COO-E polymer.

In our work, the short PDMS-COOH linear chains are crosslinked into a three-dimensional network by poly(ethylene glycol) diglycidyl ether (PEGDGE) units (Figure 1a). The PDMS-COOH, which contain abundant carboxylic acid groups along the polymer backbone, was synthesized according to our previous work [50]. The one-pot reaction between PDMS-COOH and PEGDGE with a stoichiometric ratio of 1:0.015 lead to the PDMS-COO-E polymer films, which possess abundant hydrogen bonds and permanent covalent crosslinked networks (Figure S1). The obtained PDMS-COO-E polymer is transparent and the transmittance the polymer is higher than 85% at the wavelength from 425 nm to 700 nm, which indicates that the PEMS-COO-E polymer has the potential applications in the transparent electronic devices (Figures S2 and S3).

The PDMS-COO-E polymer was characterized by FT-IR measurements (Figure S4). The FT-IR spectrum of PEGDGE shows a distinctive epoxy group absorption band at 910 cm^–1^. After reacting with PDMS-COOH at 120 °C for 4 h, the original epoxy group absorption band gradually disappeared, and a new absorption band at 1736 cm^–1^ appeared, which corresponds to the C=O stretch of the newly formed ester bond, indicating the formation of the three-dimensional network.

The thermal properties of PDMS-COO-E were investigated. According to the DSC tests (Figure 1b), the glass transition temperature (*T_g_*) of PDMS-COO-E polymer was measured at about 0.5 °C. It demonstrates that the sample of PDMS-COO-E polymer is in soft state at room temperature (>*T_g_*) with high chain mobility. The thermal stability of PDMS-COO-E polymer films were characterized by thermal gravimetric analysis (TGA) experiments in a nitrogen atmosphere (Figure 1c). Owing to the permanent three-dimensional crosslinked networks, the thermal stability of PDMS-COO-E is obviously superior to the linear polymer PDMS-COOH. Figure 1c shows that the PDMS-COO-E is thermal stable under 200 °C. Moreover, the PDMS-COO-E film has good durability toward wet environment. As shown in Figure S5, the mass of the sample remain almost unchanged during 10 days with different humidity. The good stability of this polymer should originated from the hydrophobic PDMS chain that blocks the penetration of water molecules and retards the hydrolysis of the ester bonds in the backbone. These data indicate that our polymer is stable at normal conditions and therefore potentially useful for wearable electronics and implanted biomedical devices.

### 2.2. Rheological Studies

**Figure 2:**
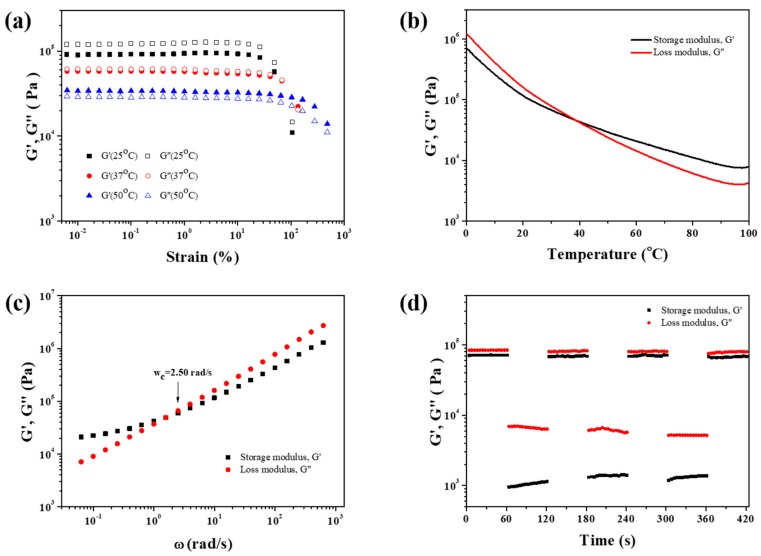
(**a**) Oscillatory strain sweeps of PDMS-COO-E at different temperatures. (**b**) Dynamic oscillatory temperature sweeps of PDMS-COO-E range from 0 °C to 100 °C at 1 Hz. (**c**) Frequency sweeps of PDMS-COO-E range from 0.01 rad/s to 1000 rad/s with 0.1% strain amplitude at room temperature. (**d**) Continuous step strain measurements of PDMS-COO-E at 25 °C and *f* = 1 Hz, under a small strain amplitude 0.1% or a large strain amplitude 500%.

Rheological properties of PDMS-COO-E polymer were systematically investigated by a rotational rheometer. According to oscillatory strain sweeps of PDMS-COO-E at different temperatures (Figure 2a), the loss modulus G′′ is higher than the storage modulus G′ when the strain is less than 25% at 25 °C, indicating that the sample is primarily viscous at comparatively small strains. When the strain increased (>25%), G′ and G′′ decreased suddenly, implying that the sample have been destroyed due to partial broken of the network. As the temperature rises to 37 °C, G′ is almost equal to G′′ at a small strain (<65%). Nevertheless, G’ is higher than G′′ at 50 °C when the strain is less than 100%, demonstrating that the material is predominantly elastic. Thus the sample transfers from viscous state into elastic state at 37 °C.

Dynamic temperature-dependent mechanical properties of PDMS-COO-E polymer were studied. Dynamic oscillatory temperature sweeps were performed ranging from 0 °C to 100 °C with a constant frequency of 1 Hz. As shown in Figure 2b, G′ of PDMS-COO-E decreases as the temperature rises. The crossover temperature of G′ and G′′ at about 37 °C indicates the viscous-elastic transformation. At about 37 °C, the hydrogen bonds between carboxyls are weak and vivid, but the three-dimensional cross-linking interactions formed by covalent bonds are still robust. Thus PDMS-COO-E is elastic as the temperature is higher than 37 °C. The rheological property of PDMS-COO-E proves the possibility of triggering the shape memory effect at body temperature.

Moreover, we performed frequency sweeps in the linear viscoelastic region of strain with 0.1% amplitude at 25 °C. In dynamic cross-linked networks, when G′ (ω) = G′′(ω), the reciprocal of the angular frequency (ω_c_), can be regarded as a characteristic relaxation time of the network (denoted as τ_c_) [51,52,53,54,55]. As illustrated in Figure 2c, the ω_c_ of PDMS-COO-E is 2.50 rad s^–1^. According to the formula τ_c_ = 1/ω_c_, the calculated relaxation time τ_c_ is equal to 0.4 s, implying ultrafast dynamic exchange of reversible hydrogen bonds in the PDMS-COO-E polymer network.

Continuous oscillation step strain experiments was conducted to monitor the recovery after mechanical breakdown. As shown in Figure 2d, the step strain measurements under the alternating small (0.1%) and larger (500%) were carried out. Above all, the typical oscillation time sweep was performed for 60 s at a small strain amplitude (0.1%) to determine the initial value of G′. When the strain increased from 0.1% to 500%, G′ decreased by 2 orders of magnitude, implying that the weak hydrogen bonds had been partially broken. When strain amplitude was brought back to 0.1%, G′ and G′′ could recovered to their original values immediately. The repeatability of this transition process was confirmed by the followed two cycles. The rapid recovery of modulus should be due to the dissociation and recombination of dynamic hydrogen bonding interactions.

### 2.3. Mechanical Measurements

**Figure 3:**
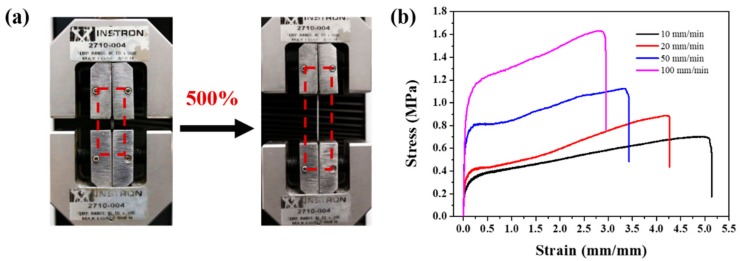
(**a**) Optical images showing the PDMS-COO-E polymer films at 0% (left) and 500% strains (right), respectively. (**b**) Stress–strain curves of the PDMS-COO-E samples with different stretch speeds range 10 to 100 mm min^–1^ at 25 °C.

The uniaxial tensile tests were performed to investigate mechanical properties of PDMS-COO-E polymer under different conditions. The stretchable PDMS-COO-E polymer films in relaxed (0% strain) and stretched (500% strain) states are shown in Figure 3a. When the strain speeds increased from 10 to 100 mm min^–1^, the breaking elongations decreased (Figure 3b) due to less time for the reform of the broken hydrogen bonds. Specifically, the PDMS-COO-E film can be stretched to 500% of its original length without fracture at 10 mm min^–1^, demonstrating the rapid rearrangement of internal polymer backbone and energy dissipation due to hydrogen bonds dissociation. When the strain rate is 100 mm min^–1^, the elongation of break decreased to 290 ± 10%.

The stretchability and mechanical strength of the resulting network can be attributed to three aspects: (i) the presence of sufficient dynamic hydrogen bonds, causes the network to stretch by an energy dissipation mechanism, (ii) the network bears a large number of high density hydrogen-bonding sites, thus leading to highly folded polymer chains, which allow easier chain sliding, and (iii) the existence of permanent covalent cross-linked networks, enhances the mechanical properties. This mechanism was further corroborated by the decrease in breaking elongations with the increasing strain rates.

Figure S6 depicts the stress relaxation curves of PDMS-COO-E polymer films at 25 °C. Obviously, owing to dynamic exchange of the dense hydrogen bonds, the normalized relaxation modulus of PDMS-COO-E rapidly decreased from 1 to 0.11 within 80 min, indicating an 89% release of the internal stress within the polymer. The relaxation at 37 °C is even faster, indicating a faster dynamic exchange of the hydrogen bonds at elevated temperature.

### 2.4. Self-Healing Properties

**Figure 4:**
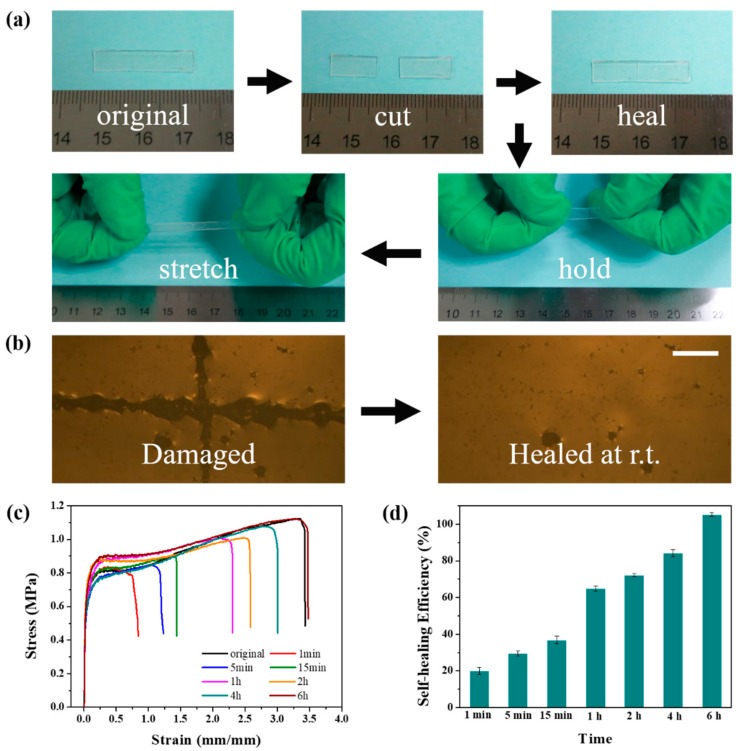
(**a**) Photographs illustrating the macroscopic cutting-healing-stretching procedure of PDMS-COO-E films at 25 °C. (**b**) Microscopic images of a film before (left) and after (right) healing at 25 °C for 6 h. Scale bars, 100 μm. (**c**) Uniaxial tensile tests of PDMS-COO-E before and after healing at room temperature for different time. (**d**) Self-healing efficiencies of PDMS-COO-E samples self-healed for different time at 25 °C.

In addition to excellent mechanical properties, the PDMS-COO-E polymer also displays a remarkable self-healing performance due to abundant hydrogen bonding. The self-healing ability of PDMS-COO-E polymer film was tested at room temperature. The sample (25 mm × 4 mm × 1 mm) was cut into two completely separate pieces with a razor blade. The two half-plates were brought back into contact at the fracture surfaces and then healed at 25 °C (Figure 4a). The self-healed PDMS-COO-E sample can be stretched to an ultimate elongation 200% of the original length after 20 min. After releasing the hands, the stretched sample can return to the initial length with a small residual strain due to the shape memory property. The cutting and healing process was observed with an optical microscope. As shown in Figure 4b, the notch on PDMS-COO-E film totally disappeared after healing for 6 h.

Furthermore, Figure 4c depicts the representative stress–strain curves for the cut PDMS-COO-E polymer film that were healed for different length of times. The mechanical self-healing efficiency (*η*) was defined as the proportion of the restored toughness (area under the stress-strain curve), which taking into account of the recovery of both stress and strain. In detail, the PDMS-COO-E film can be held and stretched after 15 min with 36% ± 2.2% self-healing efficiency. The complete healing of the cut takes 6 h at room temperature with a completely recovered ultimate elongation of 340%. The repaired PDMS-COO-E polymer film follows almost the same shape and mechanical property of the uncut sample.

### 2.5. Shape Memory Properties

**Figure 5:**
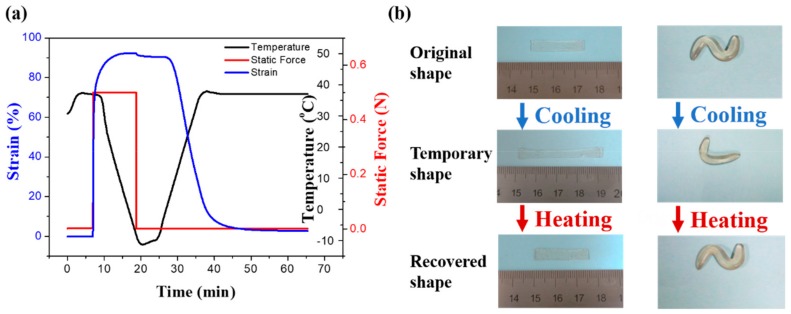
The shape memory properties of PDMS-COO-E. (**a**) Dynamic thermomechanical analysis about shape memory properties. (**b**) Macroscopic images showing the shape-memory functions of the PDMS-COO-E films.

The Dynamic thermomechanical analysis about shape memory in Figure 5a was obtained under a stress-controlled mode with identical deformation and recovery temperatures of 37 °C. In detail, the sample is in stretched state under a constant stress, then decrease the temperature (<*T_g_*) to fix the temporary shape. As the temperature rises, the shape of the PDMS-COO-E film is recovered to the permanent shape. The shape fixity ratio is 98.06% and the shape recovery ratio is 97.06% after heated for 20 min, which suggests that the material has excellent shape memory property. As shown in Figure 5b and Movie S1, the PDMS-COO-E samples can be deformed into various temporary shapes then recover the permanent shape triggered by body temperature. We also measured the shape memory property of PDMS-COO-E at 25 °C and 50 °C, respectively (Figures S7 and S8). The results show that this polymer exhibits shape memory effect at these two temperatures, but the shape recovery ratios at 25 °C (90.60%) and 50 °C (87.23%) are lower as compared to that of 37 °C.

**Figure 6:**
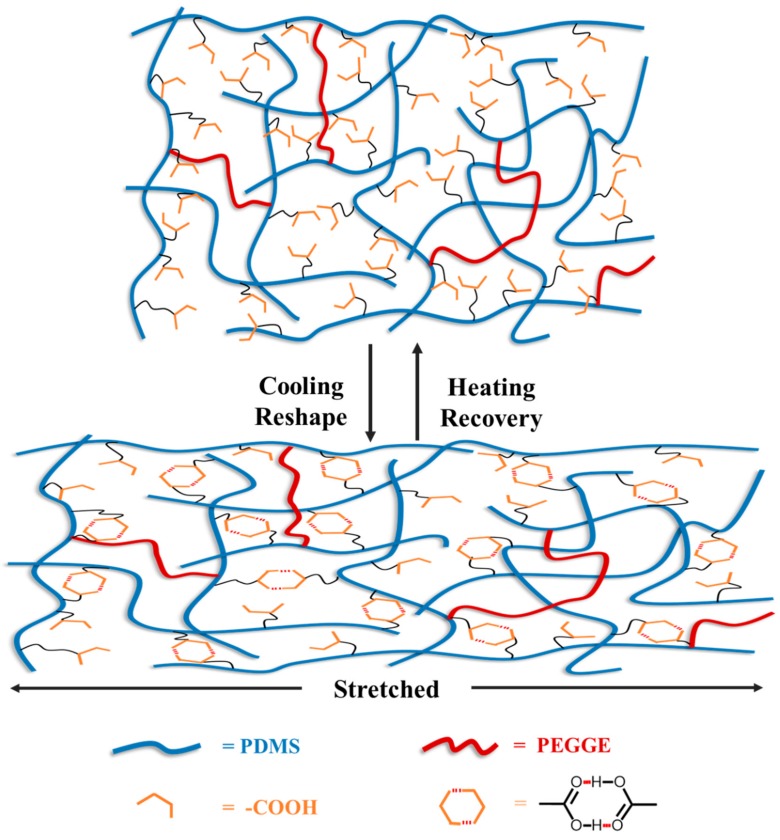
The possible mechanism about shape memory effect of PDMS-COO-E polymer.

Figure 6 illustrates the possible mechanism about shape memory effect of PDMS-COO-E polymer. The short PDMS linear chains are crosslinked by chemical covalent interactions and abundant hydrogen bonds into a three-dimensional network. In detail, the covalent cross-linked networks of PDMS-COO-E maintain the permanent shape and resilience. When the temperature is below the *T_g_* value, the samples can be molded into various temporary shapes. As the temperature rises to 37 °C, the weak hydrogen bonds are broken, and the dynamics of polymer chains increase, resulting in recovering the permanent shape. Meanwhile, a large number of hydrogen bonds enable the samples to heal at temperature without external stimulus.

## 3. Experimental Section

### 3.1. Materials

Poly(methylhydrosiloxane), trimethylsilyl terminated (PHMS, M_w_ = 1800–2100, Mole% MeHSiO = 100%) was purchased from Gelest (Morrisville, PA, USA). Methyl methacrylate (MMA), Karstedt catalyst solution (Pt, 2% in xylene), poly(ethylene glycol) diglycidyl ether (PEGDGE, M_n_ = 500), Zinc acetate, and other chemicals and solvents were purchased from Sigma-Aldrich (St. Louis, MO, USA). All of the chemicals were used as received without further purification.

### 3.2. General Measurements

FT-IR spectra were obtained by a Bruker (Billerica, MA, USA) Tensor 27 Fourier transform infrared spectrometer. Differential scanning calorimetry (DSC) experiments were conducted with a DSC apparatus of Mettler-Toledo (Zurich, Switzerland) ranging from −20 to 100 °C with a heating and cooling speed of 10 °C min^–1^ under a nitrogen atmosphere. Temperature and enthalpy calibrations were performed before the experiments using zinc and indium standards. Furthermore, each sample underwent three cooling-heating runs and the data were procured from the second cooling-heating curves. Thermal gravimetric analysis (TGA) data were recorded on the PerkinElmer (Waltham, MA, USA) TA 2100-SDT 2960 under N_2_ atmosphere. The temperature range is from 30 to 800 °C, with a heating rate of 10 °C min^–1^. Optical microscopy images were performed by Nikon (Tokyo, Japan) ECLIPSE E100 optical microscope.

### 3.3. Preparation of PDMS-COO-E Polymer Films

Synthesis of PDMS-COOH was according to the previous literature [50]. PEGDGE (0.50 g, 1 mmol) and zinc acetate catalyst (0.22 g, 1 mmol) was added to a solution of PDMS-COOH (10 g) in tetrahydrofuran (50 mL) with constantly stirring. The reaction mixture was stirred for 4 h at 120 °C in an oil bath. The resultant transparent product was dissolved in ethanol. Then, the liquid was poured into a polytetrafluoroethylene (PTFE) mold and evaporated at room temperature for 24 h followed by drying at 80 °C for 24 h. The PDMS-COO-E polymer films were peeled off from the PTFE mold and cut into specific shapes and ready for further testing.

### 3.4. Rheological Tests

The rheological measurements were performed by a rheometer (DHR-2, TA Instruments, New Castle, DE, USA). All tests were on 20 mm parallel plates with circular samples of a 20 mm diameter. The gap distance was fixed at 1000 μm. Contact force with the sample was maintained by the auto-compression feature set to 0.20 ± 0.15 N. Oscillatory strain sweeps were performed ranging from 0.01% to 1000% strain at 1 Hz at 25 °C. Temperature sweeps were conducted from 0 °C to 100 °C at a rate of 5 °C min^–1^ and a frequency of 1 Hz. The strain was automatically modulated at 0.10 ± 0.02% by the instrument to keep the measured torque at a reasonable value when the sample was softened. Frequency sweeps were run from 0.01 to 100 rad s^–1^ with a constant 0.1% strain amplitude at 25 °C.

### 3.5. Mechanical and Self-Healing Measurements

Uniaxial tensile experiments were conducted on an Instron (Boston, MA, USA) 3343 instrument equipped with a 500 N load cell at room temperature (25 °C). For all the tests, a sample size of 50 mm length × 5 mm width × 1 mm height was adopted, and each experiment was performed in triplicate. For the self-healing tests, the film was cut into two completely separate pieces and then manually put together and healed at room temperature, without applying any pressure or other external stimuli. The typical strain-stress curves were obtained by measuring the healed samples in the same procedure as the original samples. The strain rate of self-healing tests is 50 mm min^–1^. Maximal stress strengths, breaking strains, and healing efficiencies (η) are presented as the means ± standard deviation according to the data from at least four trials. Stress relaxation experiments were performed on rectangular samples (30 mm length × 4 mm width × 1 mm height) using a dynamic mechanical analysis machine (DMA, Q800, TA Instruments). The samples were equilibrated for 5 min at a specified temperature, then pulled in constant strain (50%) and maintained for 30 min. After 30 min, the stress was released and the samples were allowed to relax for an additional 90 min.

### 3.6. Shape Memory Tests

Measurements were conducted with a dynamic mechanical analysis machine (DMA, Q800, TA Instruments) in a tensile mode. All the tests adopted with a sample size of 30 mm length × 4 mm width × 1 mm height, and each measurement was performed three times.

## 4. Conclusions

In summary, we designed a polymer network with a permanent covalent crosslinking and abundant weak hydrogen bonds. The former introduces elasticity and maintains the permanent shape, and the latter contributes to the temporary shape via network rearrangement. The obtained PDMS-COO-E polymer films exhibit excellent mechanical properties and the capability to efficiently self-heal for 6 h at room temperature. Furthermore, the samples turn from a viscous state into an elastic state at 37 °C, and the resultant polymer has shape memory effects triggered by body temperature. Thus, we consider this unique material will have a wide range of applications in many fields, including wearable electronics, biomedical devices, and 4D printing. Such applications will be the subject of our future studies.

## Supplementary Materials

The following are available online. Figure S1: The schematic structure of PDMS-COO-E, Figure S2: The optical photo of PDMS-COO-E, Figure S3: UV-vis spectra of PDMS-COO-E with thickness of 0.5 mm, Figure S4: FT-IR spectra of PDMS-COOH, PEGDGE and PDMS-COO-E., Figure S5: The dependence of sample mass of PDMS-COO-E on relative humidity for 10 days, Figure S6: Stress-relaxation of PDMS-COO-E at various temperatures, Figure S7: The shape memory property of PDMS-COO-E at 25 °C, Figure S8: The shape memory property of PDMS-COO-E at 50 °C. Movie S1: The shape memory effects triggered by body temperature.
