# Supplementary material for: A Self-Healing and Shape Memory Polymer that Functions at Body Temperature
Source: Molecules. 2019 Sep 4;24(18):3224. doi: 10.3390/molecules24183224 (PMC6767172; doi:10.3390/molecules24183224)
Supplement: Supplementary file 1 [file molecules-24-03224-s001.pdf]

Supporting Information

A Self-healing and Shape Memory Polymer that Functions at Body Temperature

Hui-Ying Lai, Hong-Qin Wang, Jian-Cheng Lai and Cheng-Hui Li*

State Key Laboratory of Coordination Chemistry, School of Chemistry and Chemical Engineering, Nanjing National Laboratory of Microstructures, Collaborative Innovation Center of Advanced Microstructures, Nanjing University, Nanjing 210023, P. R. China

*Correspondence: chli@nju.edu.cn; Tel.: +86-025-8966-7389

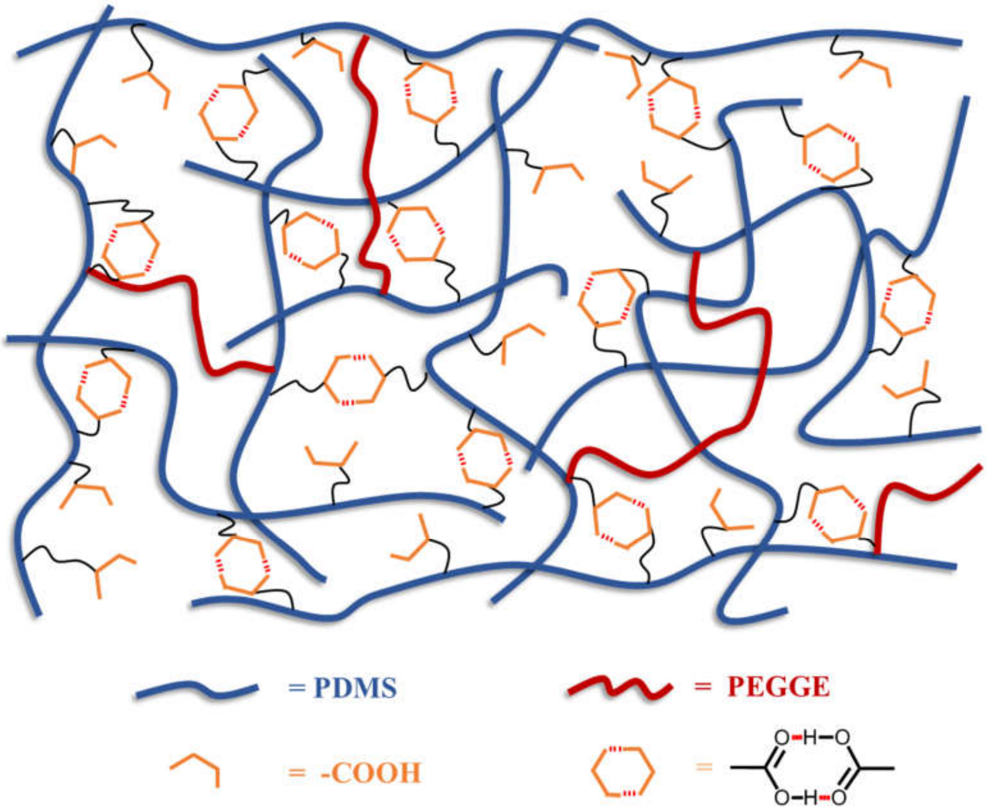

Figure 1. The schematic structure of PDMS-COO-E.

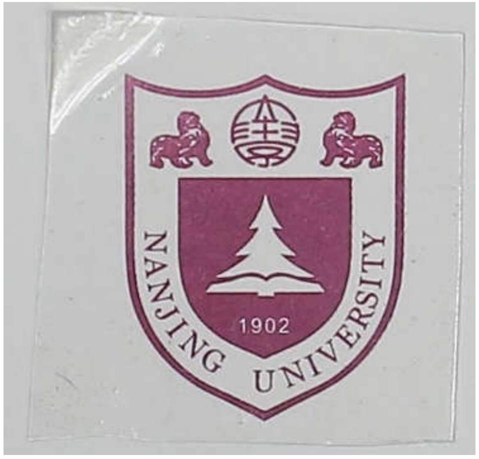

Figure 2. The optical photo of PDMS-COO-E.

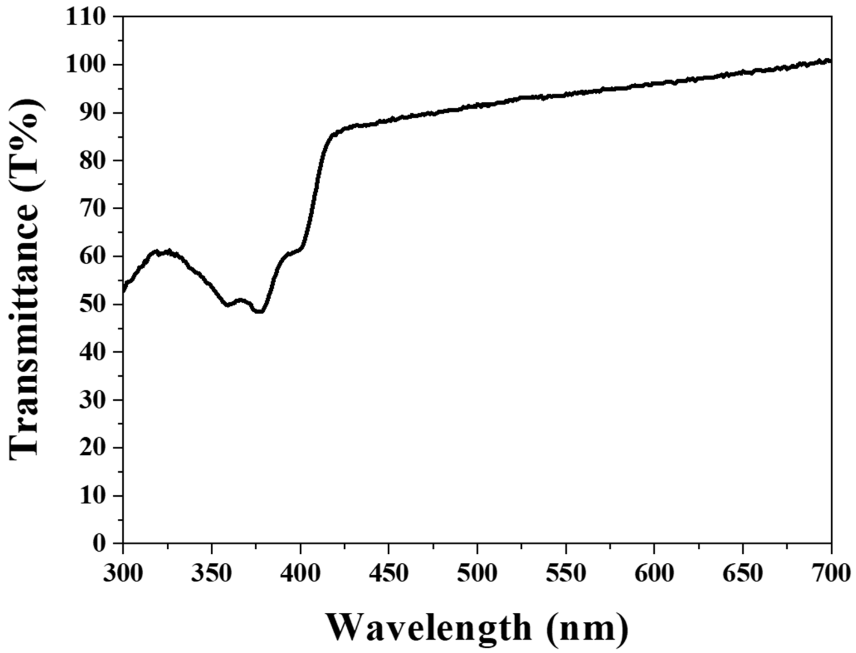

Figure 3. UV-vis spectra of PDMS-COO-E with thickness of 0.5 mm.

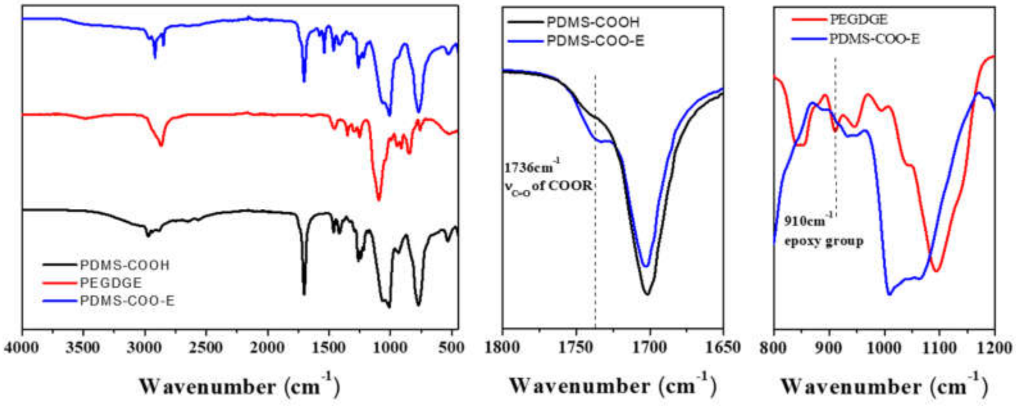

Figure 4. FT-IR spectra of PDMS-COOH, PEGDGE and PDMS-COO-E.

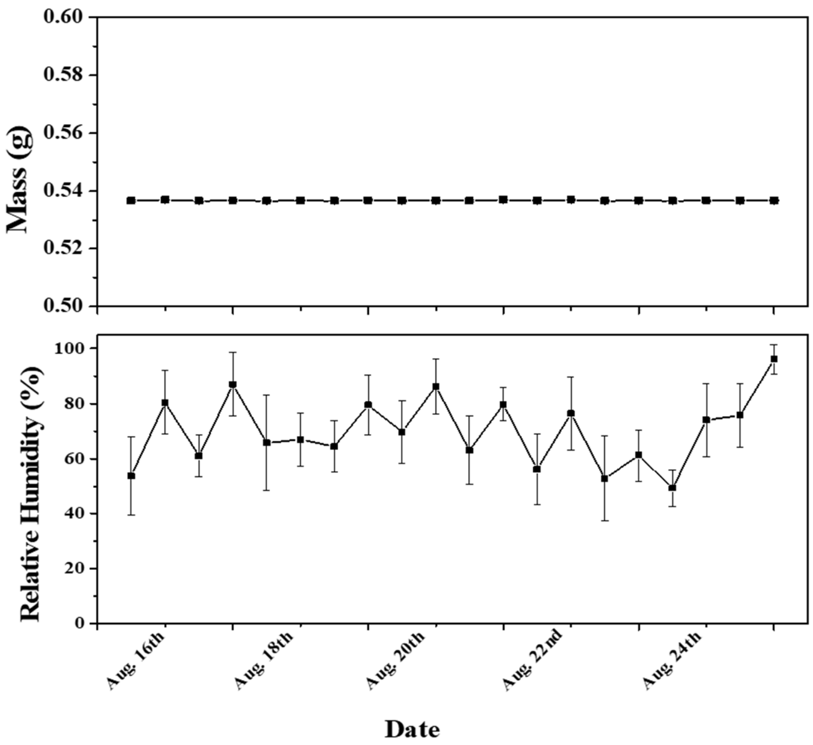

Figure 5. The dependence of sample mass of PDMS-COO-E on relative humidity for 10 days. The data were recorded every 12 h. The mass of the samples was stable with the change of humidity.

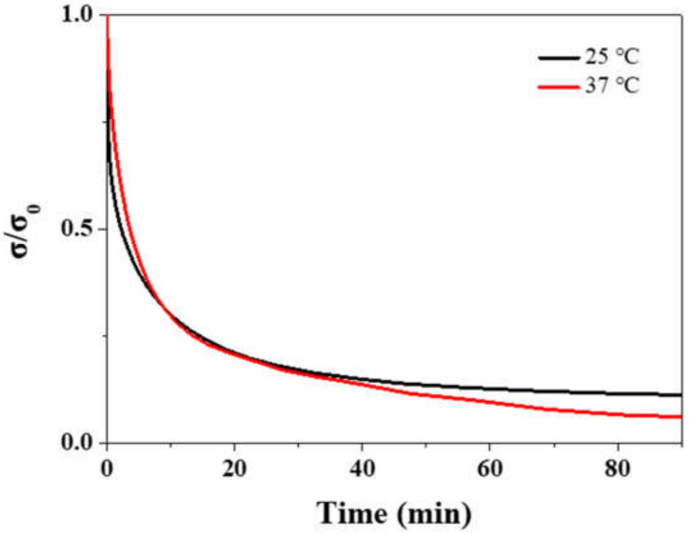

Figure 6. Stress-relaxation of PDMS-COO-E at various temperatures. The sample was pulled to reach a 50% strain, which was set at this strain for relaxation for 90 minutes.

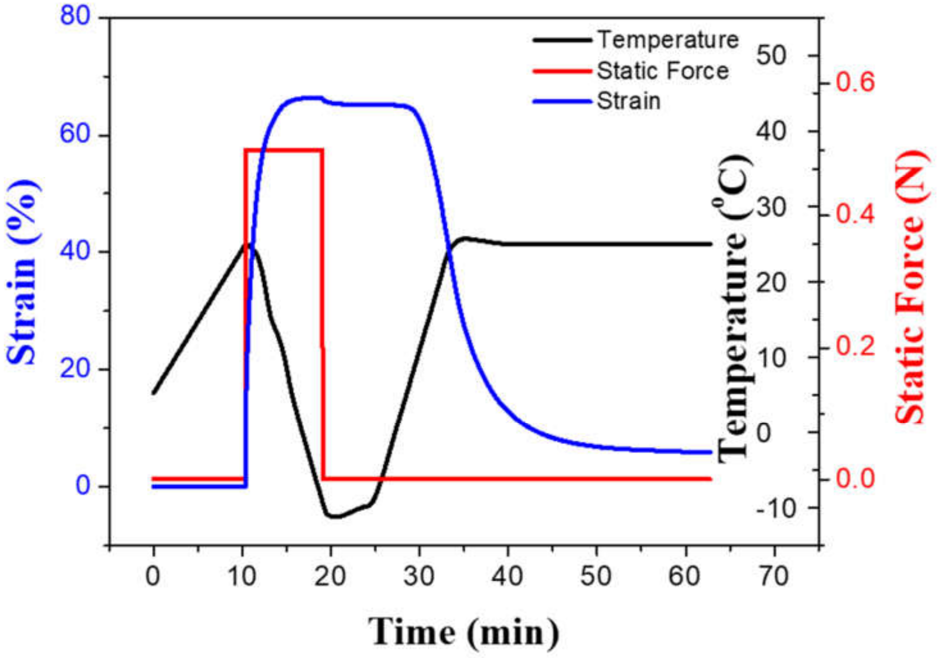

Figure 7. The shape memory property of PDMS-COO-E at 25 °C. The sample was stretched at 25 °C and fixed at -10 °C, followed by recovery at 25 °C in a stress-controlled mode. The shape fixity ratio is 98.24% and the shape recovery ratio is 90.60%.

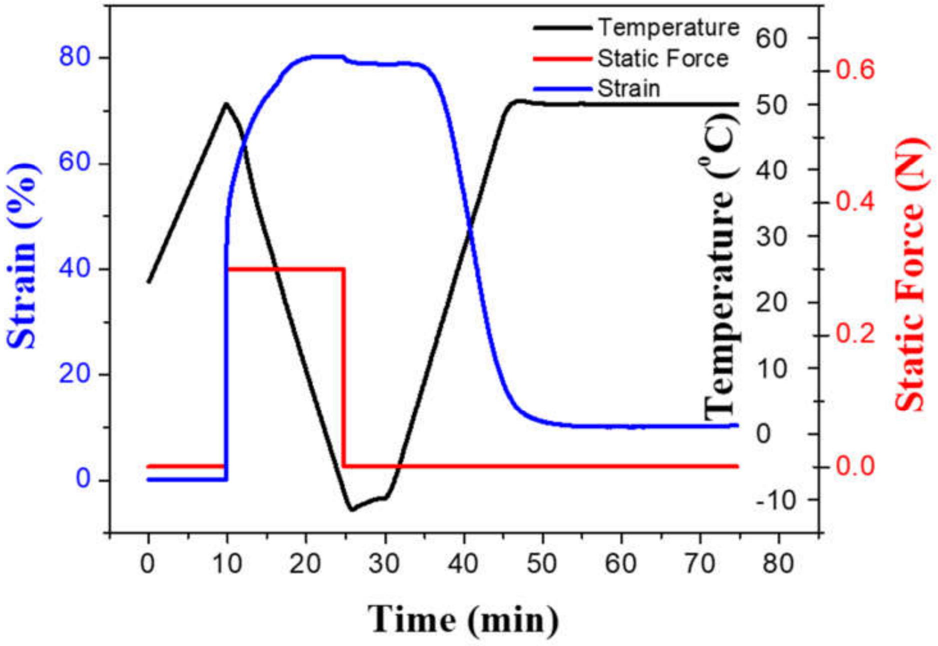

Figure 8. The shape memory property of PDMS-COO-E at 50 °C. The sample was stretched at 50 °C and fixed at -10 °C, followed by recovery at 50 °C in a stress-controlled mode. The shape fixity ratio is 98.29% and the shape recovery ratio is 87.23%.
